# Whole transcriptome RNA-Seq allelic expression in human brain

**DOI:** 10.1186/1471-2164-14-571

**Published:** 2013-08-22

**Authors:** Ryan M Smith, Amy Webb, Audrey C Papp, Leslie C Newman, Samuel K Handelman, Adam Suhy, Roshan Mascarenhas, John Oberdick, Wolfgang Sadee

**Affiliations:** 1 Department of Pharmacology, Program in Pharmacogenomics; College of Medicine, The Ohio State University Wexner Medical Center, 5184A Graves Hall, 333 West 10th Avenue, Columbus, OH 43210, USA; 2 Department of Biomedical Informatics, Program in Pharmacogenomics; College of Medicine, The Ohio State University Wexner Medical Center, Columbus, OH 43210, USA; 3 Department of Neuroscience; College of Medicine, The Ohio State University Wexner Medical Center, Columbus, OH 43210, USA; 4 Departments of Pharmacology, Psychiatry, Human Genetics/Internal Medicine, College of Medicine, College of Pharmacy, and Environmental Health Sciences, The Ohio State University Wexner Medical Center, Columbus, OH 43210, USA

**Keywords:** RNA-Seq, Whole transcriptome, Allele expression, mRNA expression, Functional genetics, Regulatory polymorphism, eQTL, Read alignment, Next generation sequencing, Bioinformatics

## Abstract

**Background:**

Measuring allelic RNA expression ratios is a powerful approach for detecting *cis*-acting regulatory variants, RNA editing, loss of heterozygosity in cancer, copy number variation, and allele-specific epigenetic gene silencing. Whole transcriptome RNA sequencing (RNA-Seq) has emerged as a genome-wide tool for identifying allelic expression imbalance (AEI), but numerous factors bias allelic RNA ratio measurements. Here, we compare RNA-Seq allelic ratios measured in nine different human brain regions with a highly sensitive and accurate SNaPshot measure of allelic RNA ratios, identifying factors affecting reliable allelic ratio measurement. Accounting for these factors, we subsequently surveyed the variability of RNA editing across brain regions and across individuals.

**Results:**

We find that RNA-Seq allelic ratios from standard alignment methods correlate poorly with SNaPshot, but applying alternative alignment strategies and correcting for observed biases significantly improves correlations. Deploying these methods on a transcriptome-wide basis in nine brain regions from a single individual, we identified genes with AEI across all regions (*SLC1A3*, *NHP2L1*) and many others with region-specific AEI. In dorsolateral prefrontal cortex (DLPFC) tissues from 14 individuals, we found evidence for frequent regulatory variants affecting RNA expression in tens to hundreds of genes, depending on stringency for assigning AEI. Further, we find that the extent and variability of RNA editing is similar across brain regions and across individuals.

**Conclusions:**

These results identify critical factors affecting allelic ratios measured by RNA-Seq and provide a foundation for using this technology to screen allelic RNA expression on a transcriptome-wide basis. Using this technology as a screening tool reveals tens to hundreds of genes harboring frequent functional variants affecting RNA expression in the human brain. With respect to RNA editing, the similarities within and between individuals leads us to conclude that this post-transcriptional process is under heavy regulatory influence to maintain an optimal degree of editing for normal biological function.

## Background

Identifying *cis*-acting functional genetic and epigenetic factors affecting RNA expression from *trans*-acting influence remains challenging. Two approaches have emerged to offset the influence of *trans*-acting factors, in search of causative *cis*-acting factors. The first approach uses large sample sizes (hundreds to thousands of tissues) to dissect the influence of *trans*- versus *cis*-acting factors influencing expression by correlating RNA transcript expression, as a quantitative trait, with single-nucleotide polymorphisms (SNP) genotyped with genome-wide arrays. Correlations between RNA expression levels and SNPs yield expression quantitative trait loci (eQTLs) located in *cis* (*cis*-eQTLs) or *trans* (*trans*-eQTLs) and have been examined across a variety of tissues [1-7]. eQTL analysis still leaves some ambiguity regarding the *cis-* or *trans*-acting nature of a polymorphism, as *cis*-acting factors can be hundreds of kilobases away from the genes they regulate [7]. As an alternative to eQTL analyses, our group and others have utilized allelic RNA expression imbalance (AEI), which compares the relative expression of two alleles in the same individual as a phenotype influenced only by *cis*-acting genetic variants [8-10]. Because AEI is an accurate and sensitive phenotype proximal to the functional genetic variant, this approach facilitates the detection of *cis*-acting regulatory polymorphisms affecting any mechanism that measurably changes RNA expression, even when those polymorphisms reside at a distance from the affected gene or in regions of high linkage disequilibrium [11]. Allelic RNA expression ratios, when measured specifically in splice variants or alternatively expressed untranslated regions, can identify genetic variants affecting RNA processing [12-14]. In addition to identifying *cis*-acting regulatory variants, AEI is a powerful phenotype for assessing the extent of RNA editing [15,16], loss-of-heterozygosity or monoallelic expression in cancer [17], and allele-specific epigenetic programming [18]. For example, directly measuring allelic-specific RNA expression in brain tumors revealed a dramatic increase in monoallelic expression of multiple oncogenes, the extent of which correlated with tumor progression and prognosis [19].

Genome-wide allelic RNA expression ratio measurements are possible by adapting genotyping array technology for quantitative measurement, demonstrating high sensitivity for detecting AEI in human cell lines and peripheral blood cells [20,21]. Multiple researchers have since used this genome-wide approach to uncover *cis*-acting regulatory variants in a variety of tissues [22-24]. However, array-based quantitative allelic analyses lacks the ability to measure AEI at rare or *de novo* SNPs and yields limited information about transcript isoform expression. The advent of massively parallel DNA sequencing technologies presents an opportunity to collect qualitative and quantitative aspects of gene expression in a single experiment, including splice isoform expression, genetic variants, *cis*-eQTLs, RNA editing, and allelic ratios [10,14,16,25-28]. However, significant experimental and analytic challenges need to be addressed and results compared to traditional methods before RNA-Seq is deemed a reliable complement (or alternative) to existing allelic measurement techniques.

Previous characterizations of allelic ratios using RNA-Seq are subject to a number of caveats, most notably high read depth requirements [29-31] and underrepresentation of variant versus reference alleles [32,33] resulting from alignment algorithms penalizing variant alleles as mismatching errors when compared to the reference genome. Bioinformatic attempts to correct variant allele underrepresentation bring allelic ratios closer to unity and result in a higher number of mapped reads [33], but may not increase reliability of allelic ratio estimates [32]. Incorporating genomic information into allelic ratio measurements, for example by normalizing allelic RNA expression ratios to matched DNA ratios or by constructing personalized reference genomes for mapping, greatly improves allelic RNA ratio estimates [14,26,27]. Still, allelic expression ratios measured by RNA-Seq are yet to be systematically compared against targeted allelic expression methods to determine the reliability of RNA-Seq to measure allelic ratios. Here, we measured allelic RNA expression ratios in 9 autopsied brain regions from a single individual, using multiple alignment strategies and comparing RNA-Seq derived allelic ratios with a highly sensitive allelic quantitation method (SNaPshot). After identifying factors affecting allelic ratio estimates by RNA-Seq, we extrapolated our methods with varying stringency to a new set of whole-transcriptome RNA-Seq samples from the dorsolateral prefrontal cortex (DLPFC) of 14 different individuals, identifying tens to hundreds of genes displaying AEI in more than one individual, indicative of frequent cis-acting regulatory variants. A number of these genes have evidence for harboring functional variants from *cis*-eQTL studies. In addition to identifying genes harboring likely *cis*-acting functional polymorphisms, we also surveyed sites of known RNA editing, asking whether we observed greater variability across brain regions in a single individual or across multiple individuals in the same brain region, shedding light on the degree to which RNA editing is regulated in the brain.

## Results and discussion

### Allelic RNA expression ratios across different alignment methods

Tissue characteristics and mapping statistics from 5500 SOLiD Sequencing (Life Technologies, Grand Island, NY) in the 9 brain tissues are presented in Table 1. We used the Ovation RNA-Seq System v2 (NuGen) for cDNA synthesis, which provides coverage at non-coding (ncRNAs) and non-polyadenylated RNA transcripts in addition to protein-coding mRNAs while reducing ribosomal RNA conversion to cDNA. Given the known alignment biases in allelic RNA expression ratios in RNA-Seq [14,26,27,32,33], we compared allelic ratios at heterozygous exonic SNPs following three different alignment methods: 1) alignment to the standard NCBI Build 37/hg19 reference genome, after which reference allele counts were directly compared to variant allele counts, 2) a targeted allele-switching method requiring construction of a new “reference” genome whereby the wild-type nucleotide at 187 SNP locations in 58 genes in NCBI Build 37/hg19 were replaced with the variant nucleotide (Additional file 1: Table S1), remapped, and corresponding “reference” allele counts from both alignments used to calculate allelic ratios, and 3) a single genome-wide alignment using a modified hg19 reference genome that incorporates International Union of Pure and Applied Chemistry (IUPAC) ambiguity codes at SNP locations catalogued in dbSNP Build 135, after which reference allele counts were directly compared to variant allele counts. Construction of an “enhanced reference genome” by adding additional loci incorporating polymorphic sites is also a viable alternative [33], as is the use of personal genomes where polymorphic sites are known [14,26], or directly sequencing the genomic DNA [27], although these were not explicitly tested here.

**Table 1:** Tissue characteristics and mapping statistics for 9 brain regions

**Brain region**	**RIN**^**a**^	**Total reads**	**Reads mapped (standard alignment)**	**Reads mapped (allele-switched alignment)**^**b**^	**Reads mapped (IUPAC alignment)**
BA10 (frontopolar cortex)	5.9	36,449,577	30,223,143 (83%)	30,222,857 (83%)	30,314,666 (83%)
BA22 (superior temporal cortex)	7.1	34,975,593	29,506,035 (84%)	29,505,770 (84%)	29,594,225 (84%)
BA24 (anterior cingulate cortex)	6.5	43,992,783	36,562,760 (83%)	36,562,530 (83%)	36,698,983 (83%)
Insular cortex	5.0	38,872,288	32,777,310 (84%)	32,777,059 (84%)	32,879,072 (84%)
Amygdala	6.9	41,172,320	34,304,449 (83%)	34,304,246 (83%)	34,398,572 (83%)
Hippocampus	6.7	48,277,234	40,944,589 (85%)	40,944,279 (85%)	41,012,249 (85%)
Putamen	6.8	30,881,648	25,861,872 (84%)	25,861,690 (84%)	25,958,094 (84%)
Cerebellum	6.9	39,047,957	32,918,344 (84%)	32,918,093 (84%)	32,938,662 (84%)
Raphe nuclei	6.9	41,231,369	33,963,780 (82%)	33,963,585 (82%)	34,091,575 (83%)

^a^*RIN* RNA Integrity Number, measured with Agilent Bioanalyzer 2100.

^b^Allele-switching only conducted on a limited number of SNPs listed in Additional file 1: Table S1.

**Figure 1:**
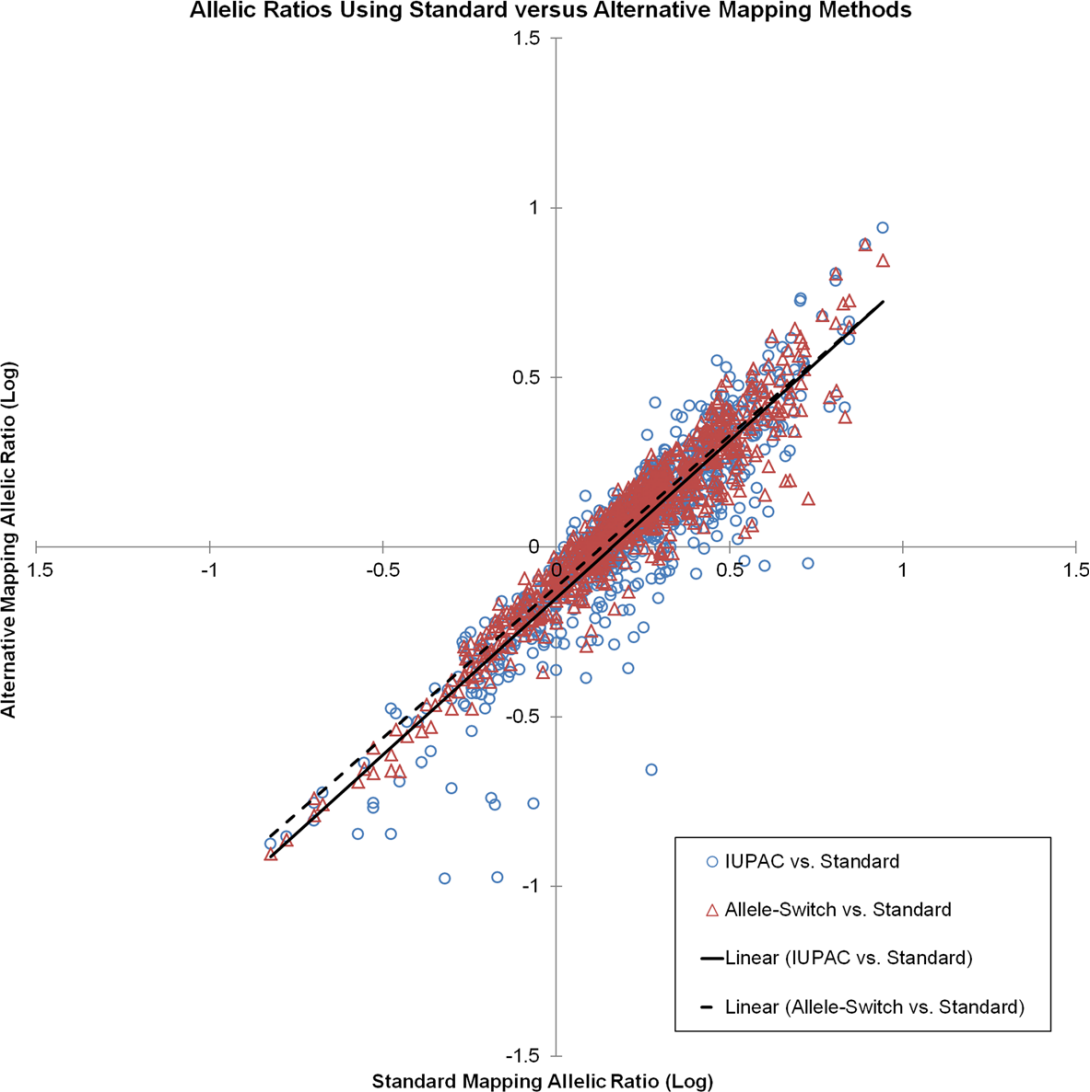
**Comparison of allelic RNA expression ratios for standard versus alternative mapping methods.** On average, the IUPAC and Allele-Switching mapping methods resulted in similar reduction of allelic RNA expression ratios (solid and dashed lines, respectively) as compared to standard mapping.

Using either alignment method that was applied in a genome-wide manner (standard or IUPAC), the number of SNPs (or genes) available for allelic ratio analysis diminishes exponentially, as higher allelic depth is required (Additional file 2: Figure S1). We limited comparisons across the three methods to those heterozygous SNPs where allelic ratio measurements were present in all three methods, for a total of 800 independent measures across 183 SNPs in 57 genes, with depth ranging from 24 to 409 reads per SNP (Additional file 3: Table S2). To estimate the magnitude of allelic ratio correction, log-transformed allelic ratios from both correction methods (IUPAC or allele-switched) were regressed against the log-transformed allelic ratios from standard alignment (Figure 1). IUPAC and allele-switch corrected data performed similarly, each reducing allelic ratio estimates compared to standard alignment (*i.e.* a 3-fold standard alignment allelic ratio corresponds to a 1.96-fold IUPAC allelic ratio and a 2.03-fold allele-switched allelic ratio).

### Allelic RNA expression ratios across cDNA synthesis methods measured with SNaPshot

The two cDNA synthesis approaches used here are methodologically different. Gene-specific priming (GSP) is a strand-specific strategy, while NuGen is strand-independent and more similar to random hexamer priming. Divergent allelic ratios between these two methods can result from an admixture of plus and minus strand-encoded RNA transcripts in the NuGen cDNA, while GSP cDNA enriches for only one strand, compelling a direct comparison between the two methods. For this comparison, we individually measured allelic RNA expression ratios at 36 different SNPs in 21 genes using SNaPshot, for a total of 186 comparisons across the 9 tissues (Additional file 4: Table S3). Overall, log-transformed allelic ratios using the two cDNA syntheses were highly correlated (*r*^*2*^ = 0.68, Additional file 2: Figure S2), although NuGen cDNA tended to yield higher allelic ratios, on average (*i.e.* a 3-fold allelic ratio in NuGen cDNA corresponds to a 2.1-fold allelic ratio in GSP cDNA). Importantly, when AEI was indicated in the GSP cDNA (>1.5-fold difference in expression between two alleles), NuGen cDNA also indicated AEI >1.5 for 17 of 21 SNPs. Similarly, when allelic ratios were <1.5 in GSP cDNA, NuGen cDNA AEI were also <1.5 at 155 of 165 SNPs. The general agreement in allelic ratios between the two cDNA synthesis methods indicates that the NuGen cDNA synthesis method used to produce the RNA-Seq libraries yields allelic ratios similar to those obtained with gene-specific priming.

### RNA-Seq allelic RNA expression ratios compared to SNaPshot

Next, we compared allelic ratios resulting from any of the three alignment methods (standard, IUPAC, allele-switch) to the single-gene SNaPshot measures of allelic expression (NuGen or GSP). Because we do not know the haplotype phasing of our samples, all allelic ratios were transformed to positive allelic ratio values using the formula |Log_10_(ratio)|, which ensures uniformity of SNPs with ambiguous strand alignment (C/G and A/T) and allows multiple allelic ratios in the same gene to be combined. For example, 2-fold and 0.5-fold allelic ratios at different SNPs in the same gene both represent a 2-fold relative difference between alleles and yield a 2-fold allelic ratio when combined within that gene.

**Figure 2:**
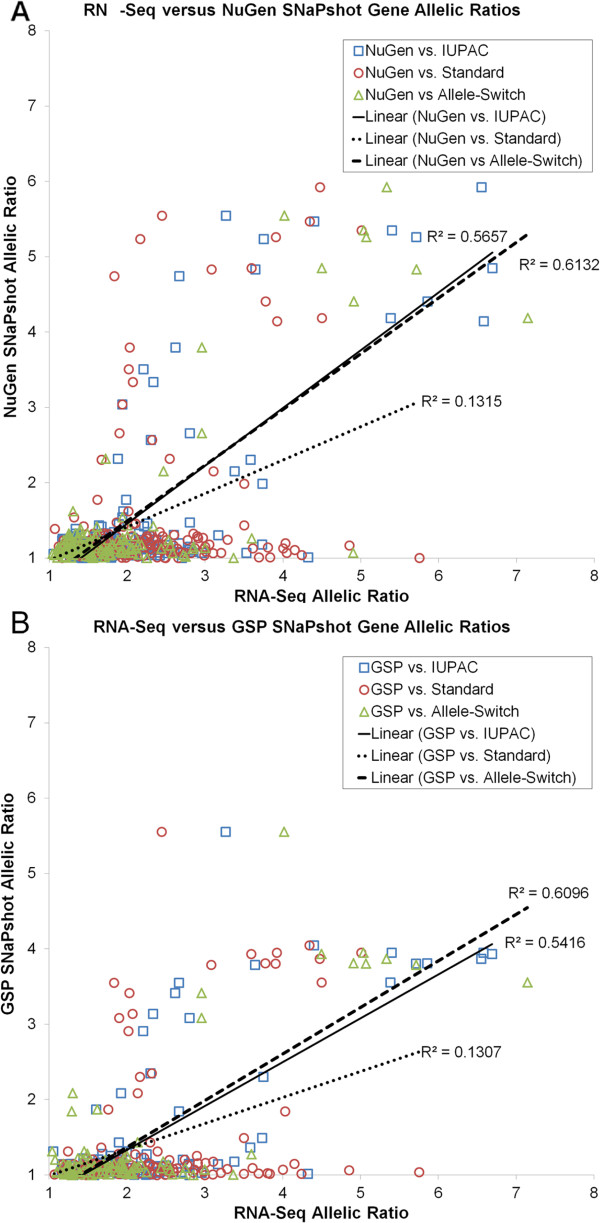
**Gene-wise allelic RNA expression ratio comparisons measured by RNA-Seq (Standard, IUPAC, or Allele-Switch) versus SNaPshot (NuGen or GSP).** The two alternative mapping methods (IUPAC and Allele-Switch) were similar and more highly correlated with SNaPshot allelic ratio measures for both NuGen cDNA **(A)** and GSP cDNA **(B)**, whereas Standard mapping was much less correlated (dotted line).

Allelic ratios, when compared at single SNPs, were similarly correlated between IUPAC- or allele-switch ratios versus either NuGen or GSP SNaPshot measures, while standard allelic ratios from RNA-Seq were much less correlated with either SNaPshot measure (Additional file 2: Figure S3). These correlations dramatically improved across all alignment methods, when allelic ratios were averaged at multiple SNPs in the same gene, although allelic ratios from the standard alignment were still much less correlated with SNaPshot measures (Figure 2).

Attempts at linear modeling, performed as a meta-analysis comparing ratios from the different methods using the metafor R package [34], did not return the theoretically expected level of agreement between observed allelic ratios from any alignment methods when compared against SNaPshot in either cDNA synthesis method (data not shown). Therefore, we considered pairwise logistic models (Additional file 2: Table S4) as an empirical meta-analytic approach for predicting whether RNA-Seq allelic ratios by any alignment method would meet a ≥1.5-fold threshold by our SNaPshot method in either NuGen or GSP libraries. Comparisons were only performed where corresponding data were present for both methods. RNA-Seq allelic ratios were converted into a logit score: log (greater number of reads / smaller number of reads), which was used as a predictor in each model [logit(AllelicRatio)]. Overall, the IUPAC alignment produced the best predictions by Akaike Information Criterion (AIC), while standard alignment performed the worst (lower scores corresponding to better model fit; Additional file 2: Table S4). For IUPAC ratios compared to SNaPshot ratios in the GSP library, the inclusion of two covariates with the logit(AllelicRatio) gave the best AIC value: the number of additional SNPs in in the gene times the logit(AllelicRatio) (as an “interaction term”) and the heterogeneity among the allelic ratios as reported by the R meta-analysis. For IUPAC ratios compared to SNaPshot ratios in the NuGen library, these covariates did not decrease the AIC, consistent with overfitting, although they also do not result in a significantly different AIC score when included in the model. Alternative approaches, including incorporation of error estimates or derivatives as covariates, did not improve the performance of the predictor. Therefore, based on logistic regression, IUPAC allelic ratios resulted in a better model fit for allelic ratios measured by SNaPshot in either cDNA synthesis method.

### Alignment correction applied across the whole transcriptome

**Figure 3:**
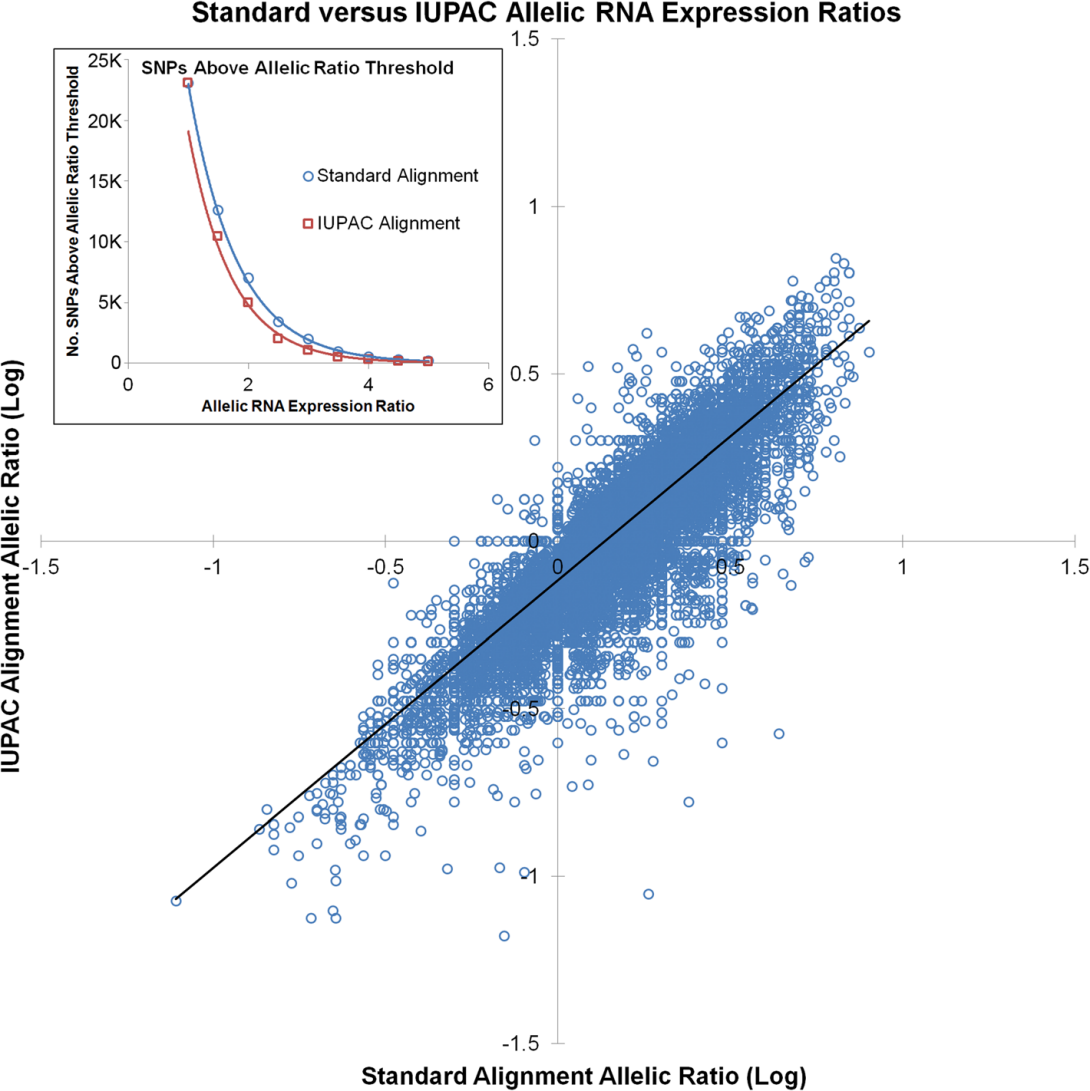
**Standard versus IUPAC-aligned allelic RNA expression ratios in all 9 brain regions.** IUPAC alignment systematically reduces allelic ratios by approximately 0.3-fold per fold change in Standard alignment allelic ratios (*i.e.* a 2-fold Standard allelic ratio corresponds to a 1.4-fold IUPAC allelic ratio). Inset: Following IUPAC alignment, fewer SNPs are above any given allelic ratio threshold compared to Standard alignment, suggesting uniform reduction of allelic ratios across the spectrum of allelic values.

Given similar ratios between the two alternative alignment methods and the results of our meta-analytic approach, we employed the IUPAC method on a transcriptome-wide basis in the 9 different brain regions (mapping statistics in Table 1). After transcriptome-wide IUPAC alignment, we compared any annotated SNP in which both alleles had a depth of at least 3 reads (6 total reads) and the lower expressed allele constituted at least 5% of total reads at the SNP, for a total of 23,085 SNPs in 3247 genes across the 9 tissues. While 6 total reads is well below the number necessary for determining statistically significant AEI at any one SNP [30], at this point we are testing the implementation of the IUPAC alignment across the entire dataset for obvious bias. The magnitude of allelic ratio correction by IUPAC alignment versus the standard alignment was similar to that observed in the smaller dataset above (*i.e.* a 3-fold uncorrected allelic ratio corresponds to a 1.96-fold IUPAC-corrected ratio; Figure 3). In the IUPAC-aligned dataset, fewer SNPs were above a given allelic ratio threshold as compared to standard alignment (Figure 3 inset), decreasing in both datasets in an exponential fashion as allelic ratios increase. This alone has significant implications for examining allelic RNA expression ratios following standard alignment. When comparing the number of SNPs displaying allelic ratios >2 between alignment methods, we see a 30-50% reduction in the number of SNPs in IUPAC versus standard alignment. Therefore, the number of SNPs displaying allelic ratios with potential biological consequence is greatly overestimated just as a consequence of standard alignment methods.

As before, we combined allelic ratios at multiple SNPs in the same gene to attenuate error in single SNP allelic ratios. Similar to the single SNP analysis, the overall number of genes displaying allelic ratios above 2 is considerably higher in the standard versus IUPAC dataset. When only requiring one SNP, 13,786 gene × tissue combinations were represented in this analysis, with allelic ratios ranging from 1- to 13.3-fold. Requiring more than one SNP per gene reduces the number of gene x tissue combinations for analysis to 4,667 and also reduces the number of genes displaying major allelic ratios greater than 5-fold (<1%). We observe 641 gene × tissue combinations with ≥2-fold allelic ratios for IUPAC alignment, as compared to 1255 combinations for standard alignment. Of the 641 IUPAC ratios ≥2, 422 (66%) were represented in the standard alignment dataset. Restricting the analysis to SNPs with at least 10 counts per allele and genes with at least 2 measurements only marginally increases coincidence of genes with allelic RNA expression ratios ≥2 between the two alignment methods (69%). So, not only do the two methods give different allelic ratio estimates, but they also produce gene pools that are only 66% similar for allelic ratios ≥2. Increasing stringency in this manner also does not appear to improve the accuracy of estimated allelic ratios when compared to SNaPshot. Five of the 26 genes that overlap between IUPAC and standard alignment (*DAD1, KCNQ3, NHP2L1, SCN1A, SCN4B*) with allelic ratios ≥2 in the IUPAC dataset were measured with SNaPshot and only one had allelic ratios ≥2 (*NHP2L1*).

The variability of allelic RNA expression ratios across multiple SNPs in a single gene is another metric that can guide our search for allelic expression imbalance. To further eliminate likely false-positives indicated by high within-gene allelic ratio variability, we can ask whether any gene-wise allelic ratio remains above a certain threshold after adjusting by the standard deviation for all SNPs within that gene. For example, 109 gene × tissue combinations (98 genes) have an allelic RNA expression ratio ≥1.5 after subtracting two standard deviations from the original allelic ratio. Of those 109 genes × tissue combinations, we measured allelic ratios in 8 using SNaPshot, 7 of which displayed allelic ratios >2. These strict requirements do exclude a number of samples where SNaPshot allelic ratios are ≥2, demonstrating the tradeoff between capturing AEI with greater probability and allowing too many false positive allelic ratios.

### Allelic RNA expression ratios across brain regions

Now that we have characterized the sensitivity of RNA-Seq in detecting allelic ratios and have estimates of the false discovery rate as compared to our single-gene methods, we can begin to provide a meaningful interpretation of the allelic ratios observed across the 9 tissues. Taking into account the factors that best improved concordance between RNA-Seq allelic ratios and those measured by SNaPshot, we used IUPAC aligned gene-wise allelic ratios averaged at multiple annotated SNPs, leaving us with the 4,667 gene x tissue allelic RNA expression ratios, 641 of which were ≥2, as noted above. We have chosen allelic ratios ≥2 in these IUPAC-aligned samples as being indicative of AEI. This allelic ratio value from IUPAC-aligned gene estimates should approximately correspond to a 1.5-fold allelic ratio by SNaPshot, according to our analysis above.

One hundred forty-nine genes had allelic ratios measurable in all 9 brain regions by our methods, none of which displayed AEI in all 9 tissues. Two genes, *NHP2L1* and *SLC1A3*, displayed AEI in 8 regions and were consistent with SNaPshot allelic ratios. High allelic differences were also observed in the ninth region for each gene, but were not included in the overall analysis as the excluded tissues had only one informative SNP for AEI measurement. *NHP2L1* encodes a protein that is a highly conserved component of the spliceosome, but the biological significance of altered mRNA expression for this gene is unknown. *SLC1A3* is the high-affinity glial glutamate transporter (also known as *Eaat1* or GLAST in rodents). Clinical phenotypes are evident for altered *SLC1A3* function, including ataxia or epilepsy, but this may be a consequence strictly resulting from protein-coding mutations, as known disease-linked mutations are presumed to act in a dominant negative fashion in the assembled homotrimeric transporter [35]. Changes only to mRNA expression, as seen in heterozygous *Slc1a3* knockout mice, do result in some behavioral abnormalities [36], but the applicability of these findings to humans is unclear. The other genes exhibiting ubiquitous AEI where measured included *ANK1, EDEM3, FAM164A, LOC338651, PTK2B, SC5DL, SEC22B, TUBA1C,* and *ZNF675*.

### Identifying common cis-acting regulatory variants in DLPFC

A primary purpose for measuring allelic ratios is to identify common *cis*-acting regulatory polymorphisms. Extrapolating our findings to a set of 14 DLPFC RNA-Seq samples from different donors, which includes the DLPFC of the donor of the other 9 brain regions, we can begin to ask which genes exhibit evidence for harboring common *cis*-acting regulatory variants and further ask which genes show the strongest evidence. Our approach includes not only protein-coding mRNAs, but also ncRNAs, which are gaining widespread appreciation for their *cis*-regulatory roles in gene expression and other important biological actions [37]. Considering the high probability that SNPs alter the conformational properties of RNA [38] and the already known importance of structure-function relationships in large classes of ncRNAs (transfer RNAs, ribosomal RNAs, *etc*.), the inclusion of ncRNAs here presents interrogation of an additional layer of *cis*-acting regulation absent when only selecting protein-coding mRNAs for RNA-Seq or subsequent analysis.

Out of 14 samples, we expect to detect functional variants with a minimum heterozygosity of ~15%, assuming that 2 samples displaying AEI in the same gene suggests a shared *cis*-acting functional variant. This analysis carries many caveats, including the assumption that only a single functional variant per gene is driving AEI, that we are able to measure allelic ratios in the same gene in all 14 samples, and that these samples do not display batch effects or artifacts associated with the library preparations or sequencing methods. Demographics and sequencing statistics are listed in Table 2.

**Table 2:** Tissue characteristics and mapping statistics for 14 dorsolateral prefrontal cortex samples

**Sample name**	**Age**	**Race**^**a**^	**Sex**	**Cocaine use**	**Smoker**	**PMI**^**b**^	**RIN**^**c**^	**Sequencing platform**	**Total reads**	**Reads mapped (IUPAC alignment)**
DLPFC1	32	AA	M	+	+	18	7.3	SOLiD 5500	94,129,888	71,736,257 (76%)
DLPFC2	49	C/H	M	+	+	15	6.6	SOLiD 5500	98,880,442	73,446,303 (74%)
DLPFC3^d^	20	AA	M	-	-	12	6.7	SOLiD 5500	92,458,538	69,487,318 (75%)
DLPFC4	34	C	M	-	+	11.5	6.8	SOLiD 5500	243,400,656	104,099,874 (43%)
DLPFC5	39	C	M	+	-	12	8.2	SOLiD 4	49,940,133	37,200,670 (74%)
DLPFC6	25	AA	M	+	-	20.5	8.2	SOLiD 4	48,399,474	37,741,267 (78%)
DLPFC7	42	C	F	+	-	14	7.1	SOLiD 4	46,956,017	35,661,219 (76%)
DLPFC8	28	C	M	-	-	11	9.1	SOLiD 4	53,947,324	39,909,693 (74%)
DLPFC9	35	C	M	-	-	12	8.3	SOLiD 4	60,022,719	44,510,882 (74%)
DLPFC10	28	C	M	+	-	8	8.1	SOLiD 4	258,721,328	200,550,177 (78%)
DLPFC11	35	C	F	+	+	11	6.4	SOLiD 4	48,215,511	37,349,598 (77%)
DLPFC12	39	C	M	-	+	11	7.7	SOLiD 4	290,364,386	227,982,821 (79%)
DLPFC13	34	C	M	-	-	22	7.3	SOLiD 4	94,036,538	56,435,396 (60%)
DLPFC14	32	B/H	F	-	-	16	9.4	SOLiD 4	48,174,311	37,197,681 (77%)

^a^Race/Ethnicity*: AA* African American, *C* Caucasian, *H* Hispanic.

^b^*PMI* Post-mortem interval, in hours.

^c^*RIN* RNA Integrity Number, measured by Agilent Bioanalyzer 2100.

^d^DLPFC originates from same sample as the 9 brain regions in Table 1.

With a cutoff of at least 3 reads per allele (6 total reads) in annotated exonic SNPs following alignment with the IUPAC reference, we calculated allelic ratios at 25,837 polymorphic sites across 7524 genes and ncRNAs in the 14 samples, for a total of 53,107 SNP x gene combinations. Using permissive parameters, we asked which genes had ≥2-fold AEI when averaged across multiple SNPs in the same gene and therefore show evidence for harboring a *cis*-acting regulatory variant. Over half (4083 of 7542) of all genes were excluded from further analysis because none of the 14 samples had more than one informative allelic ratio. In the remaining 3441 genes, we observe AEI in more than one sample for 500 genes (Additional file 5: Table S5). Specifically with respect to ncRNAs, we calculated 838 allelic ratios in 285 unique transcripts, of which, 49 exhibited allelic ratios ≥2 in two or more samples. We expect that in this analysis, we are likely overestimating the number of genes with AEI, especially given the lack of power to detect statistically significant AEI at low coverage [30]. In some cases, it is possible that AEI observed in many samples for the same gene is an artifact driven by the presence of a pseudogene or another family member with high sequence homology. For example, the gene/pseudogene *SEC22B* exhibits AEI in all 14 tissues and also displayed AEI where measured in each of the 9 brain regions. Of the 9 genes with AEI in 6 or more samples, only 2 (*ANK3* and *LMO7*) can be excluded from obvious interference by pseudogenes or highly homologous related family members. Interference from homologous family members assumes both genes are expressed in the same tissue to a level detectable by RNA-Seq and does not necessarily disqualify putative AEI without further study.

Given our permissive parameters for designating AEI and the possibility that gene homology is contributing to overrepresentation of AEI, we increased the stringency for designating AEI based on the variability of allelic ratios between SNPs in the same gene, to ask which genes have strong evidence for harboring *cis*-acting regulatory variants. As above, we subtracted two standard deviations of the within gene allelic ratios from the total gene allelic ratio for each sample and used a 1.5-fold allelic ratio as a cutoff for designating AEI. This yielded only 52 genes in which AEI was observed in multiple samples (Table 3). Three of the 52 genes were the same as those identified by the permissive analysis as having associated pseudogenes or homologous family members. Three of the 52 genes are identified as ncRNAs, but we can only exclude one ncRNA (*LINC00461*) from interference by pseudogenes or RNA editing (see below). Cross-referencing these 52 genes with *cis*-eQTLs identified by another study [1], 14 of the 44 genes where data is available exhibit evidence for harboring a common functional SNP affecting RNA expression (Table 3). As another approach, we excluded genes in which the standard deviation between SNPs in the same gene was greater than one-third of the total allelic ratio for that gene, keeping a ≥2-fold threshold for AEI. This analysis yielded 71 genes with putative AEI, 46 of which had no significant homology with the rest of the transcriptome (Additional file 6: Table S6). With respect to RNA editing described below, only *PAR-SN* and *PDIA3P* exhibited significant AEI (Table 3) and evidence for RNA editing.

**Table 3:** **Genes with AEI ≥1.5-fold following stringent correction**^**a**^

**Gene**	**# Samples measured**	**# Samples with AEI ≥1.5**	**Max AEI**^**b**^	**Min AEI**^**b**^	**Comments**^**c**^	***cis-*eQTL SNP from BrainCloud (p-val)**^**d**^
*AHNAK*	6	2	1.69	2.46	protein-coding mRNA	rs9645690 (5.94 × 10^-3^)
*ANKRD26*	5	2	1.62	1.70	pseudogene associated	rs12779247 (8.53 × 10^-8^)*
*AP2M1*	8	2	1.72	2.20	protein-coding mRNA	rs2668193 (2.77 × 10^-3^)
*AP3S2*	3	2	1.50	2.00	protein-coding mRNA	rs4932145 (4.61 × 10^-9^)*
*APOL2*	4	2	1.57	1.97	protein-coding mRNA	rs8136336 (1.03 × 10^-3^)
*ATP2B4*	8	2	1.96	2.16	protein-coding mRNA	rs3753036 (2.31 × 10^-3^)
*SESN3*	4	2	1.66	2.06	protein-coding mRNA	rs684856 (3.79 × 10^-4^)*
*C12orf5*	6	2	1.54	2.27	protein-coding mRNA	rs10849038 (4.98 × 10^-3^)
*CASD1*	6	2	1.53	1.61	protein-coding mRNA	rs2374735 (2.83 × 10^-18^)*
*CCDC25*	9	2	1.59	1.60	pseudogene associated	rs17477326 (5.35 × 10^-4^)*
*CCNT2*	6	2	1.67	1.67	pseudogene associated	rs12470730 (1.67 × 10^-2^)
*CCPG1*	8	2	1.93	2.01	protein-coding mRNA	rs11071185 (8.14 × 10^-4^)*
*CCT5*	5	2	1.61	1.77	pseudogene associated	rs606490 (5.66 × 10^-4^)*
*CLCC1*	7	2	1.67	1.70	protein-coding mRNA	rs7542414 (1.29 × 10^-3^)
*CNOT1*	7	2	1.85	1.85	protein-coding mRNA	rs11866002 (4.24 × 10^-7^)*
*DOCK4*	6	2	1.57	1.72	homology with DOCK3	rs29465 (4.47 × 10^-3^)
*EFNA5*	3	2	1.70	2.00	protein-coding mRNA	rs352602 (1.04 × 10^-4^)*
*GABRB1*	5	2	1.62	1.68	homology with other GABRB family members	N/A
*GLS*	8	2	2.22	2.29	protein-coding mRNA	rs13029532 (4.23 × 10^-2^)
*HIPK2*	10	3	1.51	2.70	ambiguity in genome assembly	rs11761839 (1.61 × 10^-2^)
*KALRN*	10	3	1.67	2.67	protein-coding mRNA	rs9873910 (1.43 × 10^-4^)*
*KCNJ16*	2	2	1.70	2.39	protein-coding mRNA	rs12940454 (1.89 × 10^-3^)
*KDM5B*	4	2	1.52	1.64	protein-coding mRNA	N/A
*KIAA1826*	4	2	1.83	2.16	protein-coding mRNA	rs2249950 (9.68 × 10^-3^)
*LINC00461*	4	2	1.62	2.50	long intergenic ncRNA	N/A
*LMO7*	10	2	1.77	2.10	protein-coding mRNA	rs1323565 (1.91 × 10^-4^)*
*LOC729799*	3	2	2.01	2.18	pseudogene associated	N/A
*MYCBP2*	7	2	1.54	1.79	protein-coding mRNA	rs1927405 (1.01 × 10^-2^)
*NGRN*	2	2	1.59	3.09	pseudogene associated	rs1543116 (4.88 × 10^-3^)
*NHP2L1*	3	2	1.56	3.21	pseudogene associated	rs17377643 (9.41 × 10^-3^)
*NIN*	9	2	1.53	2.00	protein-coding mRNA	rs10483610 (3.61 × 10^-3^)
*NIP7*	2	2	1.72	1.75	pseudogene associated	rs1127231 (2.02x10^-3^)
*NRIP3*	10	2	1.65	2.22	protein-coding mRNA	rs10840166 (1.17 × 10^-3^)
*NRSN1*	5	2	1.71	1.98	protein-coding mRNA	N/A
*OSTM1*	5	2	1.58	2.62	protein-coding mRNA	N/A
*PARG*	10	2	1.61	1.66	pseudogene associated	rs2002273 (2.18 × 10^-1^)
*PAR-SN*	2	2	2.87	3.23	imprinted and high homology with multiple genes and known RNA editing	rs2732020 (4.86 × 10^-3^)
*PDIA3P*	9	3	2.54	5.98	pseudogene associated	N/A
*PTAR1*	4	2	1.70	2.00	protein-coding mRNA	rs11139519 (4.86 × 10^-2^)
*RGMB*	4	2	1.70	2.28	protein-coding mRNA	rs2545680 (8.60 × 10^-14^)*
*RPRD1A*	4	2	1.52	1.59	pseudogene associated	rs9951407 (1.18 × 10^-2^)
*SDCCAG8*	2	2	1.67	2.12	protein-coding mRNA	rs2484639 (8.14 × 10^-6^)*
*SEC22B*	14	5	4.42	10.24	pseudogene associated	N/A
*SETD2*	3	2	1.61	1.62	protein-coding mRNA	rs1979736 (4.36 × 10^-4^)*
*SLC25A12*	3	3	1.79	2.40	protein-coding mRNA	rs4280427 (2.08 × 10^-2^)
*SPOCK2*	7	2	1.61	1.68	protein-coding mRNA	rs7894516 (6.53 × 10^-2^)
*STRBP*	3	2	1.50	1.52	protein-coding mRNA and ncRNA	rs700085 (1.50 × 10^-2^)
*SYBU*	4	2	1.81	1.87	protein-coding mRNA	rs1954713 (5.07 × 10^-2^)
*TBL1XR1*	5	2	1.61	1.93	pseudogene associated	rs6765337 (5.48 × 10^-3^)
*TP53BP1*	7	2	1.51	2.25	protein-coding mRNA	rs12912505 (1.03 × 10^-1^)
*UBB*	9	2	1.51	1.54	pseudogene associated	rs11869614 (1.03 × 10^-1^)
*WDR82*	3	2	1.53	1.92	pseudogene associated	rs730050 (1.53 × 10^-2^)

^a^Correction was performed by subtracting 2 standard deviations of the within-gene allelic ratio from the uncorrected allelic ratio.

^b^Corrected allelic ratio values reported.

^c^Pseudogene associations noted from NCBI Gene database. Homology noted where >50% coverage and >50% similarity with other annotated transcripts by Basic Local Alignment Search Tool (BLAST).

^d^Most significantly-associated SNP with any probe for the corresponding gene reported in BrainCloud [1]. N/A = no data available for gene. * = significant *cis*-eQTL association.

### Surveying RNA editing within and across brain regions

From these single base allelic ratios we can readily detect instances of RNA editing, as one type of post-transcriptional modification, and survey the variability of editing at single sites and the extent to which they are edited across different brain regions and across different individuals in the DLPFC (Table 4). In this analysis, DLPFC3 is included with the other 9 brain regions, as it originates from the same donor brain (see Table 2). We are specifically interested in determining whether RNA editing is more variable across brain regions in a single individual or within a single brain region across many individuals. Answering this question yields insight into the regulatory factors guiding this process. For example, greater variability across regions suggests that each region has a unique complement of *trans*-acting proteins guiding this process that is stable across individuals, while greater variability across individuals suggests that each region contains a common complement of *trans*-acting factors that can vary across individuals, among possible interpretations.

**Table 4:** RNA editing across brain regions and across individuals

**Gene**	**Genomic position**^**a**^	**Position in gene**	**Editing across regions**^**b**^			**Editing across individuals**^**c**^		
			***# Regions displaying editing***	***Avg. Read Depth***	***% Edited (S.E.M.)***	***# Individuals displaying editing***	***Avg. Read Depth***	***% Edited (S.E.M.)***
*CCDC75/EIF2AK2*	chr2:37327702	intergenic	8	11.4	59.8 (8.0)	8	22.2	32.0 (8.7)
*CTSB*	chr8:11702542	3′UTR	10	16.8	99.4 (0.6)	8	11.0	87.3 (4.0)
*FTX*	chrX:73499965	exonic (ncRNA)	9	62	31.8 (2.8)	10	116.3	27.1 (2.2)
*GRIA2*	chr4:158257875	exonic (non-synonymous)	10	32.3	88.9 (4.6)	13	56.0	97.5 (0.6)
*GRIA2*	chr4:158257879	exonic (synonymous)	8	35.9	21.0 (2.7)	8	75.5	16.4 (0.8)
*GRIK2*	chr6:102337689	exonic (non-synonymous)	10	19.2	44.5 (3.9)	13	18.9	37.2 (3.8)
*GRIK2*	chr6:102337702	exonic (non-synonymous)	9	18.2	62.0 (3.7)	13	17.1	63.9 (5.7)
*MTRNR2L1*	chr17:22021971	intergenic	10	1595.7	98.0 (0.1)	10	1796.7	96.9 (0.5)
*PAR-SN*	chr15:25227816	exonic (ncRNA)	9	11.6	67.8 (8.2)	13	29.6	79.4 (2.0)
*PAR-SN*	chr15:25227838	exonic (ncRNA)	7	12.1	43.5 (5.3)	11	26.1	35.4 (2.6)
*PAR-SN*	chr15:25227854	exonic (ncRNA)	5	14.4	25.4 (5.6)	8	26.6	22.1 (2.8)
*TRUB2*	chr9:131071533	3′UTR	5	11.2	54.6 (9.9)	11	15.1	59.8 (4.6)

^a^According to GRCh37/hg19.

^b^10 regions from a single individual.

^c^Only the DLPFC from 13 different individuals.

Cross-referencing our RNA-Seq data with known RNA editing sites from the DAtabase of RNa EDiting in humans (DARNED) [39], we find 2,358 and 3,249 sites noted in DARNED where we observe expression of the variant allele in the 10 different brain regions and 13 DLPFC, respectively (1,271 overlapping). We applied stringent criteria (see Methods for more information) to maximize the likelihood we are capturing true instances of RNA editing and also required at least 5 of 10 regions and 9 of 13 DLPFC to exhibit RNA editing at any one site for comparisons, leaving only 12 RNA editing sites in 8 genes (Table 4). The magnitude of editing at each of the 12 sites is highly correlated (*r*^*2*^ = 0.88) across the 10 regions and 13 DLPFC. In addition, the variability of RNA editing is not significantly different within the 10 regions as compared to the 13 DLPFC, suggesting RNA editing is tightly regulated at these 12 sites. These findings could be driven by our stringent criteria for designating RNA editing sites, but other studies have found similarly consistent levels of intra- versus inter-individual adenosine-to-inosine editing [40] and at greater depth in the brain [41].

RNA editing does impact some single-gene allelic ratio estimates. A total of 43 genes had annotated polymorphisms (with assigned rs numbers) where RNA editing is also reported in DARNED, including 13 of the 574 genes exhibiting ≥2-fold AEI in our less stringent analysis (Additional file 5: Table S5 Notes). Four of these 13 genes would not meet criteria for AEI analysis in one or more samples if the putative editing site was excluded, lacking the minimum 2 sites we required for allelic ratio estimates. Four of the 9 remaining genes have at least one sample previously exhibiting ≥2-fold AEI which now exhibits allele ratios <2-fold after excluding the putative RNA editing sites.

## Conclusions

The current study provides assessment of allelic RNA expression with RNA-Seq in human brain tissues compared to a robust and accurate SNaPshot method we routinely apply to single-gene studies of AEI. Our two cDNA synthesis methods produced similar allelic ratios, but large systematic allelic biases were evident using standard alignment methods. We find considerable variability in RNA-Seq derived allelic RNA expression ratios, but also the ability to detect AEI of various magnitudes, after adjusting for possible biases. Two factors were crucial to improve allelic RNA expression ratio measures by RNA-Seq; attenuating alignment bias and requiring more than one SNP per gene when averaging allelic ratios at regions of low depth. We tested two read alignment methods for attenuating allelic bias, each with a different strategy to account for variant alleles. Because use of the IUPAC nomenclature for biallelic SNPs yielded a closer to normal distribution of error than the other methods and was readily implemented in a single alignment strategy, we employed this technique on a transcriptome-wide basis for subsequent analyses. One potential drawback of alignment using the IUPAC reference genome is that it only provides ambiguity codes at SNPs annotated in db135 and is not usable by the widely-used Tuxedo Suite analysis package. However, Novoalign http://www.novocraft.com/main/index.php), MOSIAK (https://github.com/wanpinglee/MOSAIK/wiki/QuickStart), and MIRA (http://sourceforge.net/projects/mira-assembler/files/) are capable of alignments using genomes with ambiguity codes and are compatible with other sequencing platforms. Given the widespread recognition of allelic bias in RNA-Seq data, the approach outlined here is one of many possibilities for alleviating this artifact [14,26,27,32,33], but one particularly relevant for those utilizing LifeScope software to map and analyze reads generated by SOLiD sequencing.

By either correction method, we can substantially attenuate the reference alignment bias, showing that RNA-Seq data aligned with standard methods requires a much larger allelic ratio for confidently predicting AEI. Following standard alignment, only 468 annotated SNPs in 181 genes across all 9 tissues meet a 2.15-fold threshold (corresponding to a 1.5-fold allelic ratio following IUPAC alignment) when requiring at least 10 reads per allele. According to statistical inference, more than 100 reads are required to detect a 1.5-fold allelic ratio at *p* < 0.05 [30]. Considering that many of the genes we previously studied demonstrating phenotypic changes with allelic ratios of approximately 1.5 to 2-fold would not meet this depth requirement, this level of stringency is severely limiting but necessary if RNA-Seq is used as the sole determinant of AEI. In fact, even with permissive read requirements, brain-expressed genes we previously published as harboring *cis*-acting regulatory variants with our SNaPshot method were absent from our RNA-Seq analysis [11,13,42-46] due to a lack of coverage, with the exception of *HTR2A*[12]. This illustrates an important, albeit obvious characteristic of RNA-Seq based analyses of allelic expression – that the ability to measure allelic ratios critically depends upon read depth. At present, modest- to low-expressing genes are more effectively interrogated by other means, such as SNaPshot or highly-multiplexed amplicon resequencing [10]. Nonetheless, when used in combination with other methods, RNA-Seq is a valuable screening tool for identifying common *cis*-acting functional polymorphisms.

Our analysis of ncRNAs in this study found similar percentages of transcripts exhibiting AEI across the DLPFC as compared to protein-coding mRNAs, regardless of stringency. However, most of the ncRNAs identified in the more stringent analyses have evidence for interference by pseudogenes, with the exception of *LINC00461*. This ncRNA is up-regulated in a uniform fashion in the brain relative to the rest of the body according to microarray analysis [47,48], although a brain-specific role for this transcript is yet to be established. *LINC00461* is alternatively spliced to produce at least 4 isoforms. Encoded on the same strand in the 3′ end of three spliceoforms is microRNA 9 (miR9-2), which has been associated with neuron-specific expression and neuronal differentiation during development [49,50]. AEI at this locus could indicate differential processing of the alternative *LINC00461* isoforms or miR9-2, although these interpretations remain speculative. In either case, the widespread and high expression of this ncRNA in the brain and the role of miR9 in neuronal development impel further study.

Of particular importance to finding *cis*-acting functional variants is the ability to reliably detect AEI at single SNPs, rather than requiring averaging across multiple SNPs in the same gene, as done here. Single SNP AEI resolution allows interrogation of subtle aspects of gene regulation, if the SNP resides in a differentially processed area of the transcript, such as between two different polyadenylation signals or in an alternative exon or untranslated region [14]. In fact, our single-gene studies have now identified two regulatory SNPs that are only detected by measuring AEI in differentially expressed regions of the genes and are not apparent when measuring AEI in constitutively expressed regions of the genes [12,51]. At present, multiple factors have improved the ability to measure allelic ratios at single SNPs in differentially processed areas of a transcript, including increasing read depth and incorporating the genomic makeup of the sample into statistical analyses [14,26,27,32,33]. The sensitivity of RNA-Seq to detect AEI at single SNPs will likely continue to improve as read length increases, sequencing error rate decreases, and additional strategies are developed to account for variant alleles.

Single SNP analysis is necessary to evaluate post-transcriptional transcript modifications, such as RNA editing. Our survey of RNA editing sites in the brain is consistent with previous reports of RNA editing conducted at much greater depth [41], suggesting that single position resolution of allelic RNA expression ratios can be reliably measured at lower depth using RNA-Seq. Further, the lack of variable editing across brain tissues and across individuals argues that RNA editing in the brain is critically maintained at an optimal level, supported by observations of dysregulated RNA editing in cancer [52]. The most obvious candidates for regulating this process globally are the adenosine deaminase enzymes, *ADAR* and *ADARB1*. Although we find some correlation between mRNA expression of *ADAR* or *ADARB1* and RNA editing at these 12 sites, a more comprehensive analysis is necessary to further speculate on this relationship across the brain. While our study helps establish intra- and inter-individual differences in RNA editing in the human brain, the speed and breadth of genomic sequencing technologies is driving studies of RNA editing beyond simple quantitative levels, even revealing differences in subcellular editing events [53]. Our studies and others make it evident that RNA-Seq advances our ability to interrogate multiple aspects of the transcriptome in a single experiment, including allelic RNA expression ratios, as compared to single-gene approaches.

## Methods

### Tissue preparation and library construction

The 9 brain regions for this study were collected 12 hours post-mortem (pH 6.9) from a 20-year old African-American male smoker with no known neurological or neuropsychiatric disorders. Individual brain regions were dissected by a trained neuropathologist. The additional 14 DLPFC samples were collected in a similar manner (demographics and tissue characteristics in Table 2). RNA from each of the brain regions was TRIzol-chloroform extracted and purified with RNeasy Mini Kit spin columns (Qiagen, Germantown, MD), following standard protocol for on-column DNase treatment. DNA was isolated from the tissues using a ‘salting out’ method [54] supplemented with additional sodium dodecyl sulfate for lipid-rich brain tissue. Following nucleic acid isolation, 10 ng of total RNA was converted to cDNA using the Ovation RNA-Seq System V2 (Nugen Technologies, Inc., San Carlos, CA). This cDNA was used to construct libraries for massively parallel sequencing using the NEBNext DNA Library Prep Set for SOLiD (New England Biolabs, NEB, Ipswich, MA) and also for AEI measurements using SNaPshot, described below. Gene-specific primed cDNA used for SNaPshot was reverse transcribed from 500 ng of total RNA, using SuperScript III Reverse Transcriptase (Life Technologies).

### RNA-seq alignments and allelic counting

For the nine brain regions from the single individual, paired-end sequenced reads from a 5500 SOLiD System (LifeTechnologies) were mapped to the human genome with LifeScope Genome Analysis Software v2.5.1 (Life Technologies) using three different methods. First, all reads from each region were mapped to the NCBI Build 37/hg19 genome using the default LifeScope RNA-Seq parameters. Single nucleotide variants were identified with Samtools v0.1.16 [55], which provides a count of the aligned reads containing the reference or variant allele. Identified SNP locations were annotated based on UCSC annotation databases and dbSNP using annovar annotation software [56]. Based on annotation, each SNP was assigned to a location within a gene locus—whether exonic, intronic, intergenic, UTR, or upstream/downstream (within 1 kb of the coding region). Exonic allelic counts, including UTRs, for each polymorphic site were used to calculate allelic expression for this first alignment, yielding standard alignment AEI values. Allelic ratios for multiple polymorphisms residing within a 100 basepair window were averaged and treated as a single observation, since they likely do not represent independent observations they likely reside on the same sequenced library fragment. Second, at 187 heterozygous polymorphisms from 53 genes expressed in at least one brain tissue (800 total instances), we built a custom reference genome by replacing the reference allele with the variant SNP allele in NCBI Build 37/hg19 using the GATK FastaAlternateReference tool (http://www.broadinstitute.org/gatk/gatkdocs/org_broadinstitute_sting_gatk_walkers_fasta_FastaAlternateReferenceMaker.html) and remapped all reads using the same parameters as used for the standard reference genome. AEI was then calculated as the ratio of the reference allele count from the standard alignment versus the reference allele count at the switched alleles in the modified genome alignment. Finally, all reads from each brain region were mapped to a third genome containing IUPAC ambiguous nucleotide characters for each annotated SNP in dbSNP 135, downloaded from the UCSC Genome Browser (http://hgdownload.cse.ucsc.edu/goldenPath/hg19/snp135Mask/). IUPAC-corrected AEI was calculated as a ratio of the reference versus variant alleles. The 14 DLPFC libraries were made from cDNA synthesized by the NuGen Ovation RNA-Seq System, sequenced with the SOLiD 4 System or SOLiD 5500 System (Life Technologies), and aligned to the IUPAC references, as described above.

### AEI measurement by SNaPshot

Allelic mRNA expression ratios were measured by SNaPshot in NuGen or GSP cDNA libraries in duplicate by first PCR-amplifying a region surrounding the heterozygous SNP in both cDNA (5 ng of NuGen or 12.5 ng of GSP) and 25 ng genomic DNA (gDNA) with 2× Taq Master Mix (NEB) for 30 cycles in a 10 μl total reaction. Primers (0.3 μM) used for AEI are within a single exon to allow amplification of equivalent cDNA and gDNA molecules. Following amplification, excess single-stranded primers in the PCR reaction are digested by simultaneous Exonuclease I and Antarctic phosphatase (NEB) treatment. Subsequently, 2 μl of the amplified product is added to a 5 μl total SNaPshot reaction, consisting of 1.5 μl of SNaPshot Multiple Kit reagent (Life Technologies), 1 μl of 2 μM extension primer, and 0.5 μl of water. Extension primers for the SNaPshot reaction are immediately adjacent to the SNPs, which direct incorporation of a single fluorescent dideoxynucleotide at the SNP position in the PCR amplicons, with each nucleotide represented by a different fluorophore. Following SNaPshot, unincorporated fluorescent nucleotides are digested by Calf Intestinal Phosphatase (NEB) and the resultant fluorescent SNaPshot product is separated and detected by capillary electrophoresis on an ABI3730 DNA Analyzer. Peak heights for the different fluorescent products calculated using GeneMapper 4.0 software (Life Technologies) in cDNA and gDNA are used to calculate allelic ratios (reference/variant allele). Finally, cDNA ratios are normalized to gDNA ratios (representing a 1:1 relationship), yielding estimated allelic mRNA ratios, which indicate AEI if ratios significantly deviate from unity.

### Survey of RNA editing

Following IUPAC alignment in all tissues, sites deviating from the reference allele (*i.e.* SNPs) were cross-referenced with known RNA editing sites from DARNED [39]. To be included in the RNA editing analysis, we required at least 5 of the 10 regions and 8 of the 13 DLPFC to exhibit variant allele reads and for the average depth across the regions and individuals to be greater than 10 reads. We also excluded locations with ambiguity in mapping due to pseudogenes. After applying these filters, we observed some instances where the reference allele used for mapping was likely incorrect relative to our population (*i.e.* all samples demonstrate expression of only the variant alleles). In these instances, locations where a 95% confidence interval constructed from read count distribution across samples encompassed complete (100%) mapping to the variant allele were further excluded.

### Statistical analysis

All descriptive and correlative analyses were performed using SPSS v19.0 (IBM Corporation, Armonk, NY). Logistic and linear regression, and AIC calculations were performed using R (v2.15.2) (http://www.r-project.org/).

## Competing interests

The authors declare no competing interests.

## Authors’ contributions

RMS designed the studies, performed allelic ratio assays and statistical analyses, and wrote the manuscript. AW performed alignments, analyzed sequencing ratios, and revised the manuscript. ACP designed the studies, performed sequencing, and revised the manuscript. LCN performed sequencing, allelic ratio assays, and revised the manuscript. SKH performed statistical modeling and revised the manuscript. AS, RM, and JO performed allelic ratios assays and revised the manuscript. WS contributed to the design of the studies and edited the manuscript. All authors read and approved the final manuscript.

## Supplementary Material

Additional file 1: Table S1 A table denoting the allele-switched SNPs for alternative alignment. Click here for file

Additional file 2 Supplemental materials for Smith et al. Figures S1, S2, S3, and Table S4. Click here for file

Additional file 3: Table S2 A table with the RNA-Seq estimated allelic ratios used to compare the three different mapping methods. Click here for file

Additional file 4: Table S3 A table with the SNaPshot-measured allelic ratios used to compare NuGen versus gene-specific primed cDNA synthesis. Click here for file

Additional file 5: Table S5 A table listing all genes with allelic ratios ≥2 in the 14 DLPFC samples following IUPAC alignment. Click here for file

Additional file 6: Table S6 Genes exhibiting AEI using stringent correction for within-sample variability. Click here for file

## References

[B1] Colantuoni C Lipska BK Ye T Hyde TM Tao R Leek JT Colantuoni EA Elkahloun AG Herman MM Weinberger DR Kleinman JE Temporal dynamics and genetic control of transcription in the human prefrontal cortex Nature 2011 478 519 523 10.1038/nature10524 22031444 PMC3510670

[B2] Dixon AL Liang L Moffatt MF Chen W Heath S Wong KC Taylor J Burnett E Gut I Farrall M Lathrop GM Abecasis GR Cookson WO A genome-wide association study of global gene expression Nat Genet 2007 39 1202 1207 10.1038/ng2109 17873877

[B3] Gibbs JR van der Brug MP Hernandez DG Traynor BJ Nalls MA Lai SL Arepalli S Dillman A Rafferty IP Troncoso J Johnson R Zielke HR Ferrucci L Longo DL Cookson MR Singleton AB Abundant quantitative trait loci exist for DNA methylation and gene expression in human brain PLoS Genet 2010 6 e1000952 10.1371/journal.pgen.1000952 20485568 PMC2869317

[B4] Kwan T Benovoy D Dias C Gurd S Provencher C Beaulieu P Hudson TJ Sladek R Majewski J Genome-wide analysis of transcript isoform variation in humans Nat Genet 2008 40 225 231 10.1038/ng.2007.57 18193047

[B5] Morley M Molony CM Weber TM Devlin JL Ewens KG Spielman RS Cheung VG Genetic analysis of genome-wide variation in human gene expression Nature 2004 430 743 747 10.1038/nature02797 15269782 PMC2966974

[B6] Myers AJ Gibbs JR Webster JA Rohrer K Zhao A Marlowe L Kaleem M Leung D Bryden L Nath P Zismann VL Joshipura K Huentelman MJ Hu-Lince D Coon KD Craig DW Pearson JV Holmans P Heward CB Reiman EM Stephan D Hardy J A survey of genetic human cortical gene expression Nat Genet 2007 39 1494 1499 10.1038/ng.2007.16 17982457

[B7] Schadt EE Molony C Chudin E Hao K Yang X Lum PY Kasarskis A Zhang B Wang S Suver C Zhu J Millstein J Sieberts S Lamb J GuhaThakurta D Derry J Storey JD Avila-Campillo I Kruger MJ Johnson JM Rohl CA van Nas A Mehrabian M Drake TA Lusis AJ Smith RC Guengerich FP Strom SC Schuetz E Rushmore TH Mapping the genetic architecture of gene expression in human liver PLoS Biol 2008 6 e107 10.1371/journal.pbio.0060107 18462017 PMC2365981

[B8] Johnson AD Zhang Y Papp AC Pinsonneault JK Lim JE Saffen D Dai Z Wang D Sadee W Polymorphisms affecting gene transcription and mRNA processing in pharmacogenetic candidate genes: detection through allelic expression imbalance in human target tissues Pharmacogenet Genomics 2008 18 781 791 10.1097/FPC.0b013e3283050107 18698231 PMC2779843

[B9] Wang D Sadee W Searching for polymorphisms that affect gene expression and mRNA processing: example ABCB1 (MDR1) AAPS J 2006 8 E515 E520 10.1208/aapsj080361 17025270 PMC2761059

[B10] Xu X Wang H Zhu M Sun Y Tao Y He Q Wang J Chen L Saffen D Next-generation DNA sequencing-based assay for measuring allelic expression imbalance (AEI) of candidate neuropsychiatric disorder genes in human brain BMC Genomics 2011 12 518 10.1186/1471-2164-12-518 22013986 PMC3228908

[B11] Smith RM Alachkar H Papp AC Wang D Mash DC Wang JC Bierut LJ Sadee W Nicotinic alpha5 receptor subunit mRNA expression is associated with distant 5′ upstream polymorphisms Eur J Hum Genet 2011 19 76 83 10.1038/ejhg.2010.120 20700147 PMC2995013

[B12] Smith RM Papp AC Webb A Ruble CL Munsie LM Nisenbaum LK Kleinman JE Lipska BK Sadee W Multiple Regulatory Variants Modulate Expression of 5-Hydroxytryptamine 2A Receptors in Human Cortex Biol Psychiatry 2013 73 546 554 10.1016/j.biopsych.2012.09.028 23158458 PMC3582836

[B13] Zhang Y Bertolino A Fazio L Blasi G Rampino A Romano R Lee ML Xiao T Papp A Wang D Sadee W Polymorphisms in human dopamine D2 receptor gene affect gene expression, splicing, and neuronal activity during working memory Proc Natl Acad Sci USA 2007 104 20552 20557 10.1073/pnas.0707106104 18077373 PMC2154469

[B14] Li G Bahn JH Lee JH Peng G Chen Z Nelson SF Xiao X Identification of allele-specific alternative mRNA processing via transcriptome sequencing Nucleic Acids Res 2012 40 e104 10.1093/nar/gks280 22467206 PMC3401465

[B15] Bahn JH Lee JH Li G Greer C Peng G Xiao X Accurate identification of A-to-I RNA editing in human by transcriptome sequencing Genome Res 2012 22 142 150 10.1101/gr.124107.111 21960545 PMC3246201

[B16] Peng Z Cheng Y Tan BCM Kang L Tian Z Zhu Y Zhang W Liang Y Hu X Tan X Guo J Dong Z Bao L Wang J Comprehensive analysis of RNA-Seq data reveals extensive RNA editing in a human transcriptome Nat Biotechnolin press 10.1038/nbt.2122 22327324

[B17] Tuch BB Laborde RR Xu X Gu J Chung CB Monighetti CK Stanley SJ Olsen KD Kasperbauer JL Moore EJ Broomer AJ Tan R Brzoska PM Muller MW Siddiqui AS Asmann YW Sun Y Kuersten S Barker MA De La Vega FM Smith DI Tumor transcriptome sequencing reveals allelic expression imbalances associated with copy number alterations PLoS One 2010 5 e9317 10.1371/journal.pone.0009317 20174472 PMC2824832

[B18] Yang F Babak T Shendure J Disteche CM Global survey of escape from X inactivation by RNA-sequencing in mouse Genome Res 2010 20 614 622 10.1101/gr.103200.109 20363980 PMC2860163

[B19] Walker EJ Zhang C Castelo-Branco P Hawkins C Wilson W Zhukova N Alon N Novokmet A Baskin B Ray P Knobbe C Dirks P Taylor MD Croul S Malkin D Tabori U Monoallelic expression determines oncogenic progression and outcome in benign and malignant brain tumors Cancer Res 2012 72 636 644 10.1158/0008-5472.CAN-11-2266 22144470

[B20] Ge B Pokholok DK Kwan T Grundberg E Morcos L Verlaan DJ Le J Koka V Lam KC Gagne V Dias J Hoberman R Montpetit A Joly MM Harvey EJ Sinnett D Beaulieu P Hamon R Graziani A Dewar K Harmsen E Majewski J Goring HH Naumova AK Blanchette M Gunderson KL Pastinen T Global patterns of cis variation in human cells revealed by high-density allelic expression analysis Nat Genet 2009 41 1216 1222 10.1038/ng.473 19838192

[B21] Palacios R Gazave E Goni J Piedrafita G Fernando O Navarro A Villoslada P Allele-specific gene expression is widespread across the genome and biological processes PLoS One 2009 4 e4150 10.1371/journal.pone.0004150 19127300 PMC2613524

[B22] Gao C Devarajan K Zhou Y Slater CM Daly MB Chen X Identifying breast cancer risk loci by global differential allele-specific expression (DASE) analysis in mammary epithelial transcriptome BMC Genomics 2012 13 570 10.1186/1471-2164-13-570 23107584 PMC3532379

[B23] Liu R Maia AT Russell R Caldas C Ponder BA Ritchie ME Allele-specific expression analysis methods for high-density SNP microarray data Bioinformatics 2012 28 1102 1108 10.1093/bioinformatics/bts089 22355082

[B24] Morcos L Ge B Koka V Lam KC Pokholok DK Gunderson KL Montpetit A Verlaan DJ Pastinen T Genome-wide assessment of imprinted expression in human cells Genome Biol 2011 12 R25 10.1186/gb-2011-12-3-r25 21418647 PMC3129675

[B25] Majewski J Pastinen T The study of eQTL variations by RNA-seq: from SNPs to phenotypes Trends Genet 2011 27 72 79 10.1016/j.tig.2010.10.006 21122937

[B26] Rozowsky J Abyzov A Wang J Alves P Raha D Harmanci A Leng J Bjornson R Kong Y Kitabayashi N Bhardwaj N Rubin M Snyder M Gerstein M AlleleSeq: analysis of the allele-specific expression and binding in a network framework Mol Syst Biol 2011 7 522 21811232 10.1038/msb.2011.54 PMC3208341

[B27] Skelly DA Johansson M Madeoy J Wakefield J Akey JM A powerful and flexible statistical framework for testing hypotheses of allele-specific gene expression from RNA-seq data Genome Res 2011 21 1728 1737 10.1101/gr.119784.110 21873452 PMC3202289

[B28] Wahlstedt H Daniel C Ensterö M Öhman M Large-scale mRNA sequencing determines global regulation of RNA editing during brain development Genome Res 2009 19 978 986 10.1101/gr.089409.108 19420382 PMC2694479

[B29] Heap GA Yang JH Downes K Healy BC Hunt KA Bockett N Franke L Dubois PC Mein CA Dobson RJ Albert TJ Rodesch MJ Clayton DG Todd JA van Heel DA Plagnol V Genome-wide analysis of allelic expression imbalance in human primary cells by high-throughput transcriptome resequencing Hum Mol Genet 2010 19 122 134 10.1093/hmg/ddp473 19825846 PMC2792152

[B30] Nothnagel M Wolf A Herrmann A Szafranski K Vater I Brosch M Huse K Siebert R Platzer M Hampe J Krawczak M Statistical inference of allelic imbalance from transcriptome data Hum Mutat 2011 32 98 106 10.1002/humu.21396 21120951

[B31] Pickrell JK Marioni JC Pai AA Degner JF Engelhardt BE Nkadori E Veyrieras JB Stephens M Gilad Y Pritchard JK Understanding mechanisms underlying human gene expression variation with RNA sequencing Nature 2010 464 768 772 10.1038/nature08872 20220758 PMC3089435

[B32] Degner JF Marioni JC Pai AA Pickrell JK Nkadori E Gilad Y Pritchard JK Effect of read-mapping biases on detecting allele-specific expression from RNA-sequencing data Bioinformatics 2009 25 3207 3212 10.1093/bioinformatics/btp579 19808877 PMC2788925

[B33] Satya RV Zavaljevski N Reifman J A new strategy to reduce allelic bias in RNA-Seq readmapping Nucleic Acids Res 2012 40 e127 10.1093/nar/gks425 22584625 PMC3439884

[B34] Veichtbauer W Conducting meta-analyses in R with the metafor package J Stat Softw 2010 36 1 48

[B35] Jen JC Wan J Palos TP Howard BD Baloh RW Mutation in the glutamate transporter EAAT1 causes episodic ataxia, hemiplegia, and seizures Neurology 2005 65 529 534 10.1212/01.WNL.0000172638.58172.5a 16116111

[B36] Karlsson RM Tanaka K Saksida LM Bussey TJ Heilig M Holmes A Assessment of glutamate transporter GLAST (EAAT1)-deficient mice for phenotypes relevant to the negative and executive/cognitive symptoms of schizophrenia Neuropsychopharmacol 2009 34 1578 1589 10.1038/npp.2008.215 PMC3400972 19078949

[B37] Guttman M Rinn JL Modular regulatory principles of large non-coding RNAs Nature 2012 482 339 346 10.1038/nature10887 22337053 PMC4197003

[B38] Johnson AD Trumbower H Sadee W RNA structures affected by single nucleotide polymorphisms in transcribed regions of the human genome Webmed Central Bioinformatics 2011 2 WMC001600

[B39] Kiran AM O’Mahony JJ Sanjeev K Baranov PV Darned in 2013: inclusion of model organisms and linking with Wikipedia Nucleic Acids Res 2013 41 Database issue D258 261 23074185 10.1093/nar/gks961 PMC3531090

[B40] Greenberger S Levanon EY Paz-Yaacov N Barzilai A Safran M Osenberg S Amariglio N Rechavi G Eisenberg E Consistent levels of A-to-I RNA editing across individuals in coding sequences and non-conserved Alu repeats BMC Genomics 2010 11 608 10.1186/1471-2164-11-608 21029430 PMC3091749

[B41] Zhu H Urban DJ Blashka J McPheeters MT Kroeze WK Mieczkowski P Overholser JC Jurjus GJ Dieter L Mahajan GJ Rajkowska G Wang Z Sullivan PF Stockmeier CA Roth BL Quantitative analysis of focused A-to-I RNA editing sites by ultra-high-throughput sequencing in psychiatric disorders PLoS One 2012 7 e43227 10.1371/journal.pone.0043227 22912834 PMC3422315

[B42] Lim JE Papp A Pinsonneault J Sadee W Saffen D Allelic expression of serotonin transporter (SERT) mRNA in human pons: lack of correlation with the polymorphism SERTLPR Mol Psychiatry 2006 11 649 662 10.1038/sj.mp.4001797 16432527

[B43] Lim JE Pinsonneault J Sadee W Saffen D Tryptophan hydroxylase 2 (TPH2) haplotypes predict levels of TPH2 mRNA expression in human pons Mol Psychiatry 2007 12 491 501 17453063 10.1038/sj.mp.4001923 PMC1896269

[B44] Pinsonneault JK Han DD Burdick KE Kataki M Bertolino A Malhotra AK Gu HH Sadee W Dopamine transporter gene variant affecting expression in human brain is associated with bipolar disorder Neuropsychopharmacol 2011 36 1644 1655 10.1038/npp.2011.45 PMC3138671 21525861

[B45] Pinsonneault JK Papp AC Sadee W Allelic mRNA expression of X-linked monoamine oxidase a (MAOA) in human brain: dissection of epigenetic and genetic factors Hum Mol Genet 2006 15 2636 2649 10.1093/hmg/ddl192 16893905

[B46] Zhang Y Wang D Johnson AD Papp AC Sadee W Allelic expression imbalance of human mu opioid receptor (OPRM1) caused by variant A118G J Biol Chem 2005 280 32618 32624 10.1074/jbc.M504942200 16046395

[B47] Roth RB Hevezi P Lee J Willhite D Lechner SM Foster AC Zlotnik A Gene expression analyses reveal molecular relationships among 20 regions of the human CNS Neurogenetics 2006 7 67 80 10.1007/s10048-006-0032-6 16572319

[B48] Yanai I Benjamin H Shmoish M Chalifa-Caspi V Shklar M Ophir R Bar-Even A Horn-Saban S Safran M Domany E Lancet D Shmueli O Genome-wide midrange transcription profiles reveal expression level relationships in human tissue specification Bioinformatics 2005 21 650 659 10.1093/bioinformatics/bti042 15388519

[B49] Ko MH Kim S Hwang DW Ko HY Kim YH Lee DS Bioimaging of the unbalanced expression of microRNA9 and microRNA* during the neuronal differentiation of P19 cells FEBS J 2008 275 2605 2616 10.1111/j.1742-4658.2008.06408.x 18410378

[B50] Liu J Githinji J Mclaughlin B Wilczek K Nolta J Role of miRNAs in neuronal differentiation from human embryonic stem cell-derived neural stem cells Stem Cell 2012 8 1129 1137 10.1007/s12015-012-9411-6 PMC4559349 23054963

[B51] Wang D Para MF Koletar SL Sadee W Human N-acetyltransferase 1 *10 and *11 alleles increase protein expression through distinct mechanisms and associate with sulfamethoxazole-induced hypersensitivity Pharmacogenet Genomics 2011 21 652 664 10.1097/FPC.0b013e3283498ee9 21878835 PMC3172334

[B52] Paz N Levanon EY Amariglio N Heimberger AB Ram Z Constantini S Barbash ZS Adamsky K Safran M Hirschberg A Krupsky M Ben-Dov I Cazacu S Mikkelsen T Brodie C Eisenberg E Rechavi G Altered adenosine-to-inosine RNA editing in human cancer Genome Res 2007 17 1586 1595 10.1101/gr.6493107 17908822 PMC2045141

[B53] Chen L Characterization and comparison of human nuclear and cytosolic editomes Proc Natl Acad Sci USA 2013 110 E2741 2747 10.1073/pnas.1218884110 23818636 PMC3718162

[B54] Miller SA Dykes DD Polesky HF A simple salting out procedure for extracting DNA from human nucleated cells Nucleic Acids Res 1988 16 1215 10.1093/nar/16.3.1215 3344216 PMC334765

[B55] Li H Handsaker B Wysoker A Fennell T Ruan J Homer N Marth G Abecasis G Durbin R The Sequence Alignment/Map format and SAMtools Bioinformatics 2009 25 2078 2079 10.1093/bioinformatics/btp352 19505943 PMC2723002

[B56] Wang K Li M Hakonarson H ANNOVAR: functional annotation of genetic variants from high-throughput sequencing data Nucleic Acids Res 2010 38 e164 10.1093/nar/gkq603 20601685 PMC2938201

