# Supplementary material for: Whole transcriptome RNA-Seq allelic expression in human brain
Source: BMC Genomics. 2013 Aug 22;14:571. doi: 10.1186/1471-2164-14-571 (PMC3765493; doi:10.1186/1471-2164-14-571)
Supplement: Additional file 2 — Supplemental materials for Smith et al. Figures S1, S2, S3, and Table S4. [file 1471-2164-14-571-S2.pdf]

Figure S1.

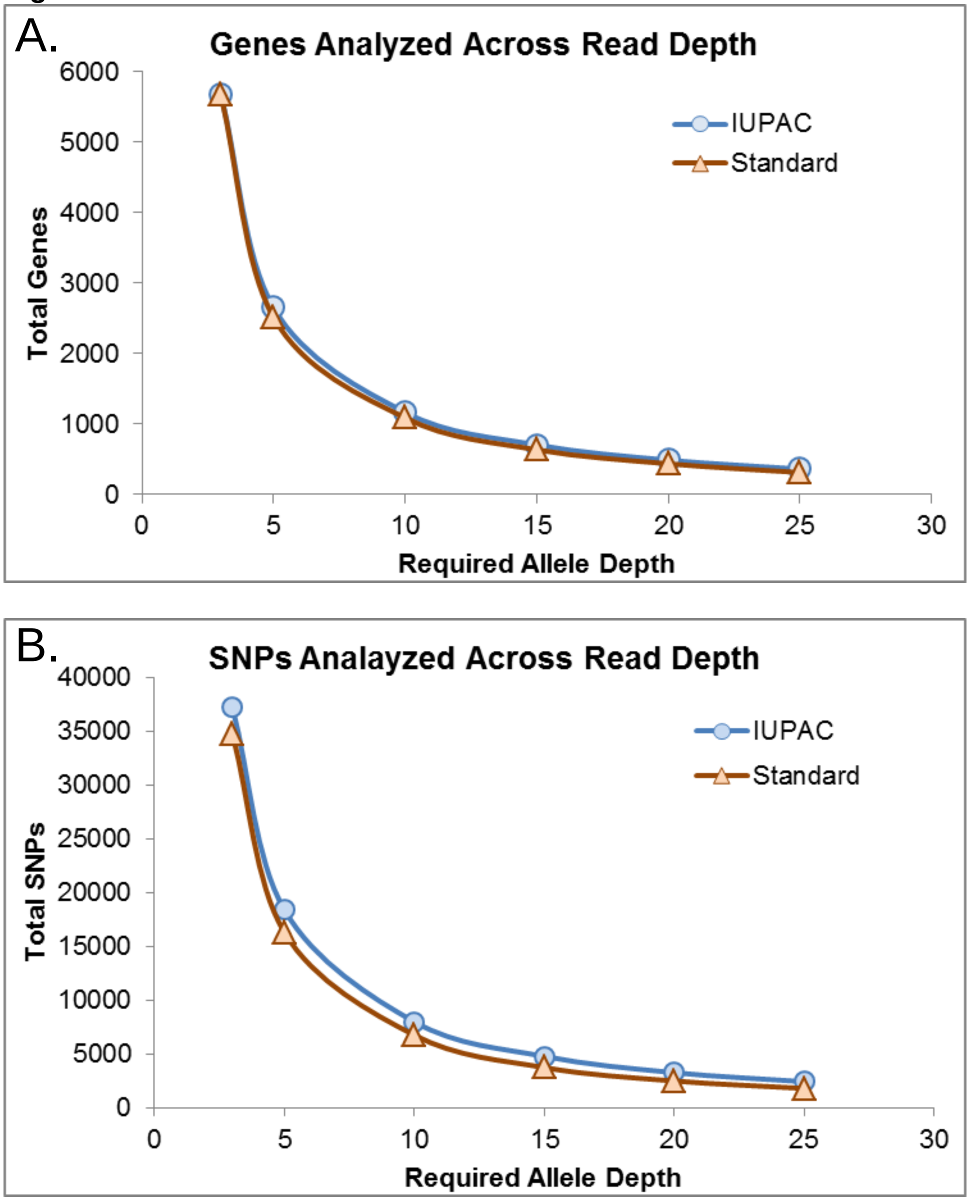

Figure S1. Total number of genes (A) or SNPs (B) available for allelic RNA expression ratio analysis at varying read depths in standard uncorrected alignments or IUPAC-corrected alignments. As more reads per allele are required, the total number of genes or SNPs available for analysis decreases at a similar exponential rate using either alignment method.

Figure S2.

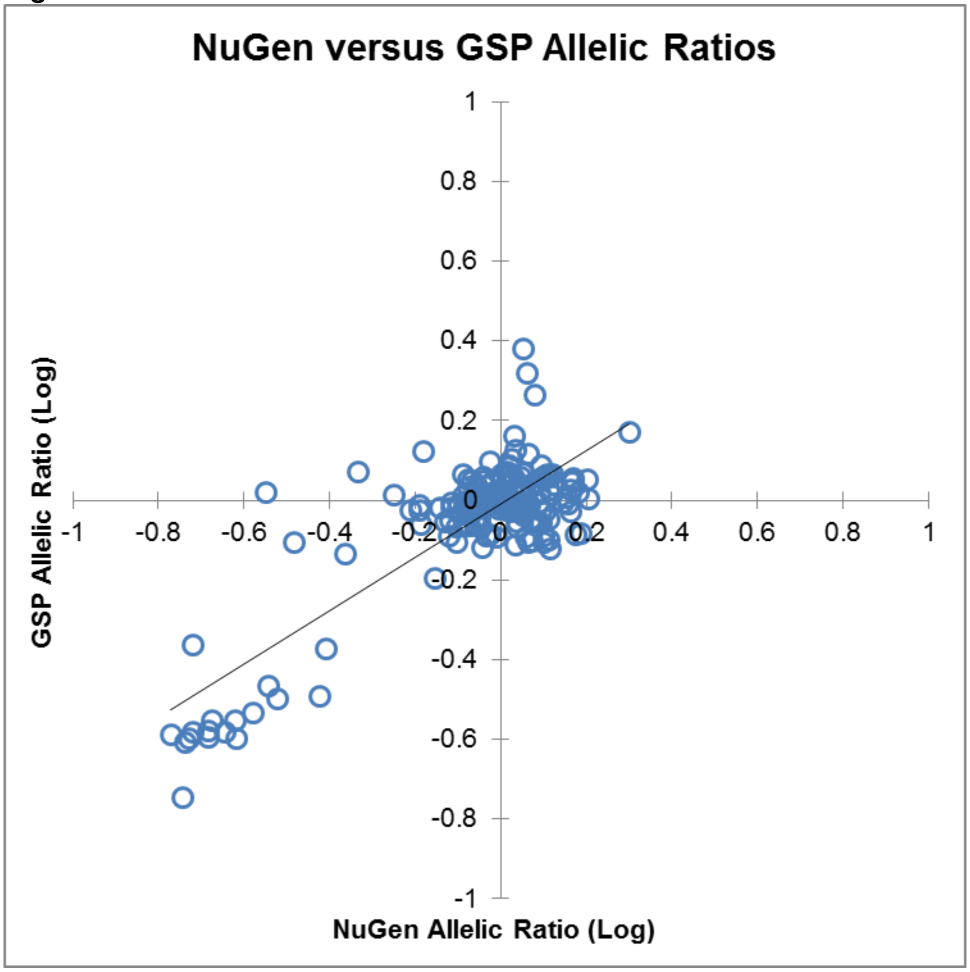

Figure S2. Comparison between allelic RNA expression ratios in NuGen versus Gene-Specific Primed (GSP) cDNA measured by SNaPshot. Overall, ratios were highly correlated (r^2=0.68) between the two difference cDNA synthesis methods.

Figure S3

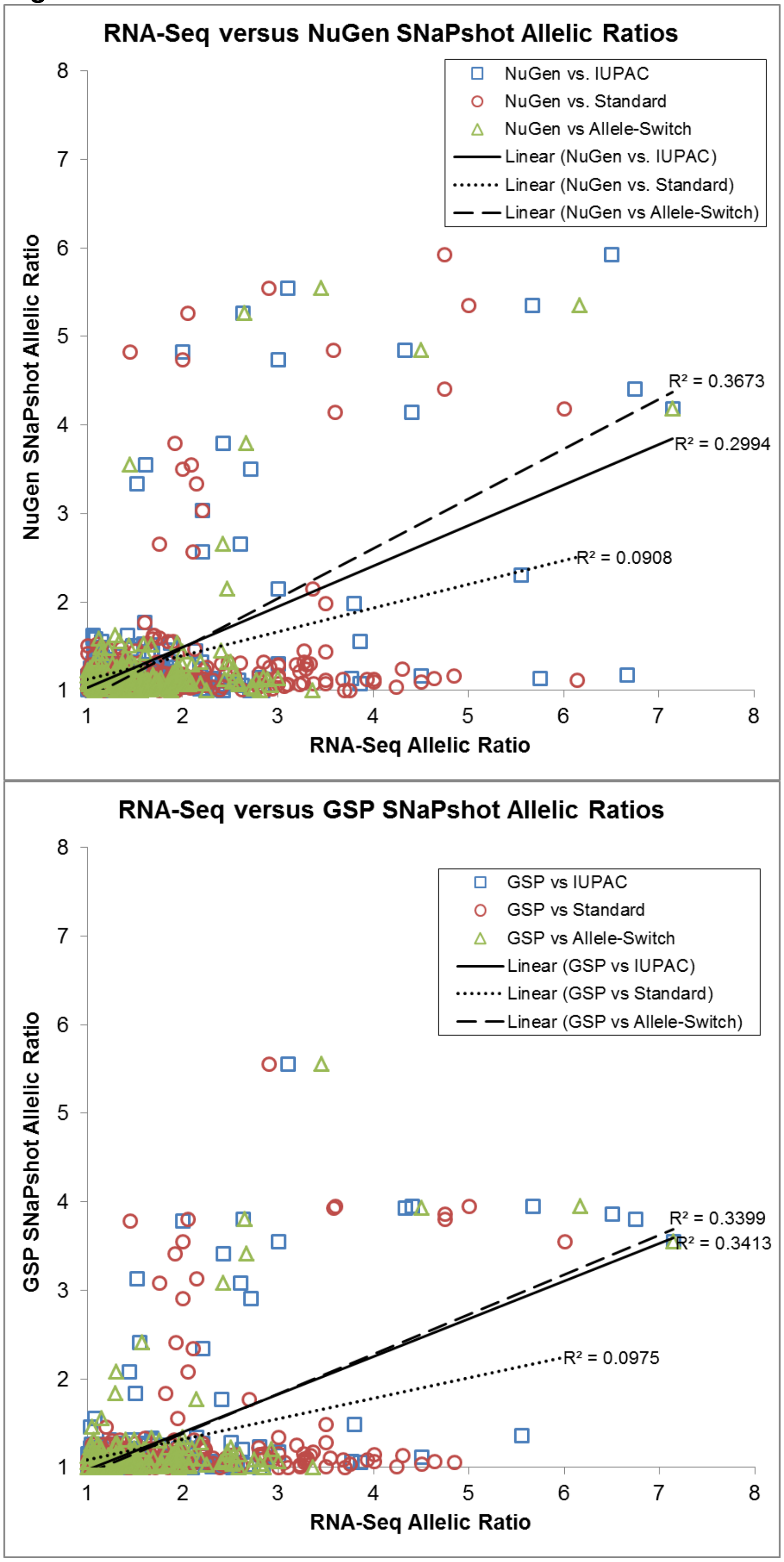

Figure S3. Single SNP comparisons between allelic ratios measured by RNA-Seq (Standard, IUPAC, or Allele-Switched alignments) versus SNaPshot (NuGen or GSP). Allelic ratios measured by SNaPshot in either cDNA synthesis method were similarly correlated to IUPAC or Allele-Switched allelic ratios, while allelic ratios from Standard alignment were much less correlated with either SNaPshot measure.

Table S4. Logistic Regression Meta-Analytic Comparisons for RNA-Seq versus SNaPshot

SNaPshot Dataset	RNA-Seq Dataset	Base Logit	β[logit(Allelic Ratio)]	β[# of SNPs]	β[Heterogeneity]	AIC
GSP	IUPAC	-3.916	2.241	-5.06 + 9.177 × logit(Allelic Ratio)	-1.511	**43.67**
GSP	IUPAC	-5.45556	4.45077		0.02694	49.69
GSP	IUPAC	-3.989	2.336	-4.03 + 5.912 × logit(Allelic Ratio)		44.22
GSP	IUPAC	-5.427	4.443			47.7
NuGen	IUPAC	-5.304	3.8846	-1.6335 + 3.0783 × logit (Allelic Ratio)	-0.2071	50.9
NuGen	IUPAC	-6.5721	5.59965		0.09292	49.93
NuGen	IUPAC	-5.344	3.928	-1.716 + 2.881 × logit (Allelic Ratio)		49.07
NuGen	IUPAC	-6.426	5.523			**48.05**
GSP	Standard	-2.9389	1.3665	-0.7606 + 1.1834 × logit (Allelic Ratio)	-0.2092	72.89
GSP	Standard	-3.71	2.165			70.06
NuGen	Standard	-3.0469	1.4264	-0.7713 + 0.9113 × logit (Allelic Ratio)	-0.1327	84.6
NuGen	Standard	-4.026	2.285			81.07
GSP	Allele-Switch	-4.5515	3.1746	-2.2551 + 5.0235 × logit (Allelic Ratio)	-0.8264	48.77
GSP	Allele-Switch	-5.196	4.204			49.34
NuGen	Allele-Switch	-5.2518	3.9856	-1.6903 + 3.2015 × logit (Allelic Ratio)	-0.2701	51.91
NuGen	Allele-Switch	-6.047	5.128			49.66
